# Successful repair of spontaneous uterine rupture in the second trimester of pregnancy with progression to live birth

**DOI:** 10.1515/crpm-2025-0025

**Published:** 2026-05-26

**Authors:** Deniz Karçaaltıncaba, Hasan Hüseyin Uçkan

**Affiliations:** Department of Obstetrics and Gynecology, Gazi University, Ankara, Türkiye; Aydın City Hospital, Aydın, Türkiye

**Keywords:** uterine rupture, cesarean section, second trimester, case report, conservative management

## Abstract

**Objectives:**

Uterine rupture before the onset of labor is a rare but life-threatening obstetric emergency. This condition carries a high risk of maternal and fetal morbidity and mortality and requires prompt and careful management.

**Case presentation:**

A 26-year-old woman, gravida 3 and parity 2, was admitted to the gynecology department with complaints of abdominal pain in her 23rd week of pregnancy. During evaluation, a decrease in hemoglobin levels was noted, and abdominal MRI revealed uterine rupture. Uterine rupture was surgically repaired with a double-layer suture with fetal preservation. Following uterine repair, the pregnancy was successfully prolonged by more than 10 weeks and the patient delivered a healthy newborn at 33 weeks and 6 days.

**Conclusions:**

Early diagnosis and appropriate surgical intervention allowed the continuation of pregnancy and delivery at 33 weeks and 6 days resulting in favorable neonatal outcomes in this high-risk case. Uterine rupture in the mid-trimester increases the complexity of management, as traditional intervention strategies may need to be adapted to ensure both maternal and fetal safety. This case supports conservative surgical repair as a potential management option in carefully selected cases of mid-trimester uterine rupture.

## Introduction

Uterine rupture is a rare but critical obstetric complication characterized by the complete tearing of the uterine wall, leading to potential catastrophic outcomes for both the mother and the fetus. Although uterine rupture is a well-known complication of labor dystocia [1], spontaneous uterine rupture outside labor is exceedingly rare. It poses significant risks, particularly in pregnancies complicated by prior uterine surgeries [2], 3].

In recent decades, the global rate of cesarean delivery has increased significantly. Also, second-trimester cesarean delivery has been used more commonly with the development of neonatal care in preterm and previable newborns. These procedures often require a thick transverse incision before development of the lower uterine segment or a classical incision, which increases the risk of spontaneous uterine rupture in subsequent pregnancies. Traditional management of uterine rupture includes emergent delivery with uterine repair when feasible or hysterectomy. Uterine rupture in the mid-trimester increases the complexity of management, as traditional intervention strategies may need to be adapted to ensure both maternal and fetal safety.

We describe a case of successful repair of a spontaneous uterine rupture at 23 weeks of gestation, which led to a live birth, and discuss the relevant literature surrounding this rare complication. This report aims to contribute to the growing pool of knowledge on this subject by reviewing published literature and highlighting evolving management strategies for these rare cases.

## Case presentation

A 26-year-old woman, gravida 3 and parity 2, presented at 23 weeks of gestation with complaints of periumbilical abdominal pain. Her obstetric history was significant for two previous preterm cesarean deliveries due to severe hypertension (at 24 weeks) and severe preeclampsia (at 29 weeks). She conceived naturally 6 months after her last cesarean section. Both previous cesarean deliveries had been performed via transverse uterine incisions; however, due to the preterm gestational age, the incisions were located outside the well-developed lower uterine segment. Physical examination revealed no abdominal tenderness or rebound.

The pelvic examination was normal with no vaginal bleeding. Transvaginal ultrasonography revealed a cervical length of 35 mm without any funneling or fluid collection in the cul-de-sac space. The obstetric ultrasound examination revealed a single, viable fetus with an estimated fetal weight of 750 g and normal amniotic fluid volume. The placenta was located on the anterior uterine wall above the lower uterine segment. Abdominal sonography was normal for the appendix, kidneys, gallbladder, and liver. Laboratory tests showed a hemoglobin level of 11.5 g/dL and a white blood cell count of 13,100 cells/mm^3^.

After 12 h of observation, she was discharged following spontaneous resolution of pain. Two days later, she was admitted to the emergency department. Upon admission, her blood pressure was 100/60 mmHg, and her heart rate was 103 bpm. The patient also reported nausea without vomiting. Abdominal examination revealed generalized abdominal tenderness and rebound tenderness, with no vaginal bleeding and no uterine contractions. Cervical length measurement was the same as that at the previous hospitalization; obstetric ultrasonography identified normal amniotic fluid volume and fetal cardiac activity. Laboratory tests showed a hemoglobin level of 10 g/dL and a white blood cell count of 16,260 cells/mm^3^.

**Figure 1:**
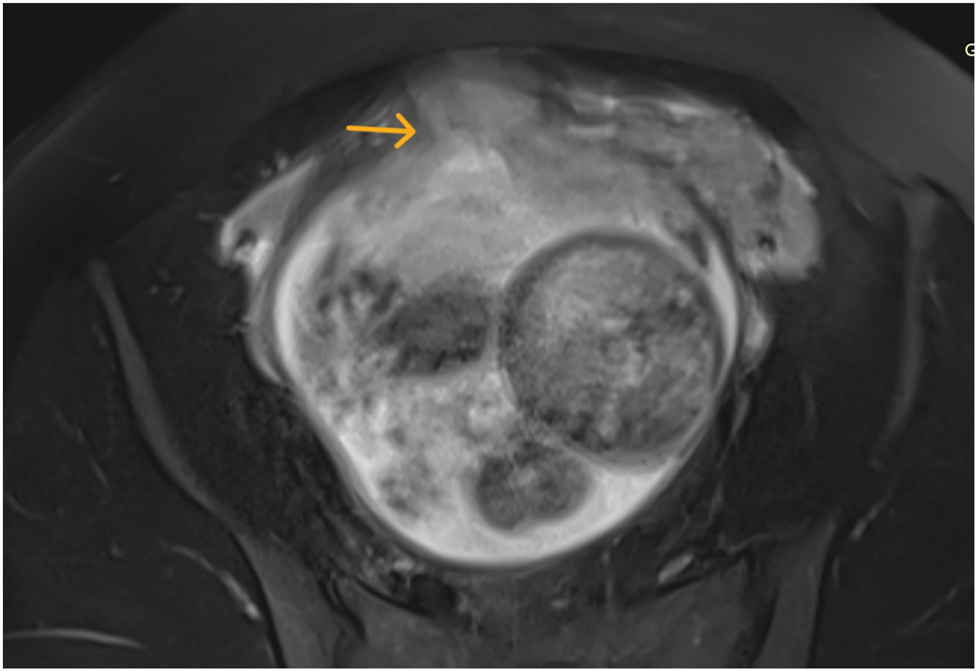
T1-weighted axial MRI showing hemorrhagic changes at the uterine rupture site.

**Figure 2:**
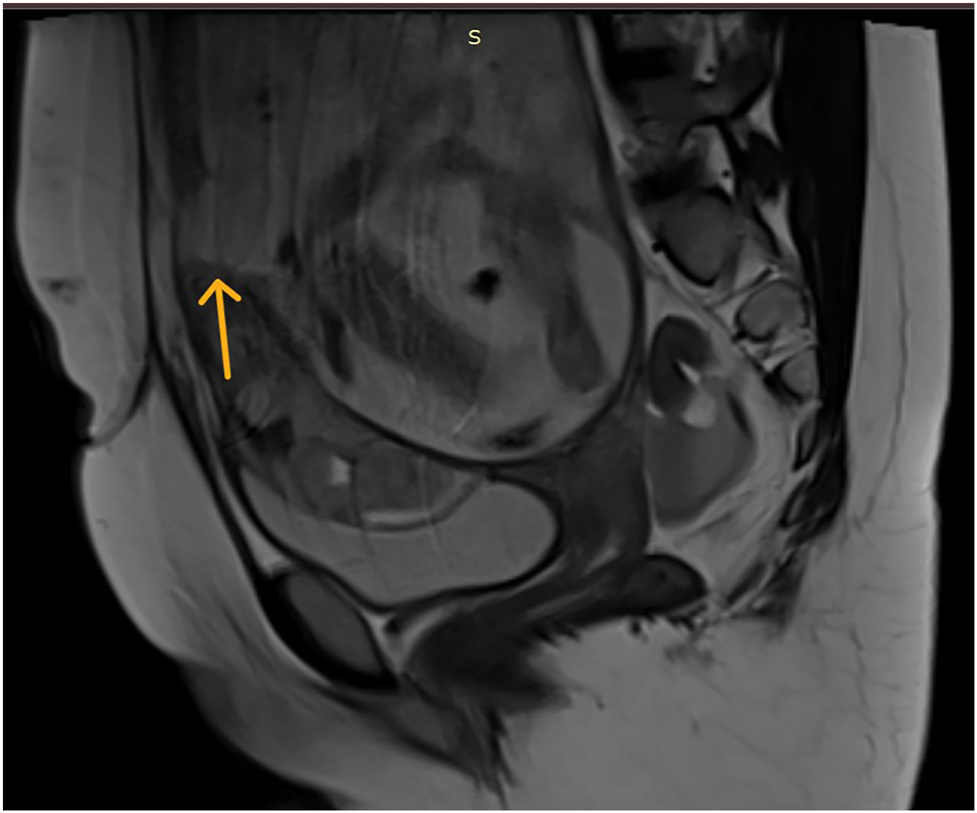
T1-weighted sagittal MRI showing hemorrhagic changes at the uterine rupture site.

Despite persistent symptoms, follow-up abdominal ultrasonography revealed no specific pathological findings. Given the severity of acute abdominal pain, abdominal MRI was performed. MRI was preferred due to its superior soft tissue contrast and absence of ionizing radiation in this hemodynamically stable pregnant patient. T1-weighted fat-saturated sagittal MRI image showed hemorrhagic changes at the contained rupture site (Figures 1 and 2). The placenta was noted to terminate just superior to the rupture site, and no sonographic or MRI findings suggestive of placenta accreta spectrum were identified, which allowed conservative surgical repair. The hemoglobin concentration decreased to 9.1 g/dL. Given the concerning physical examination, laboratory results, and MRI findings, the decision was made to proceed with diagnostic laparoscopy.

**Figure 3:**
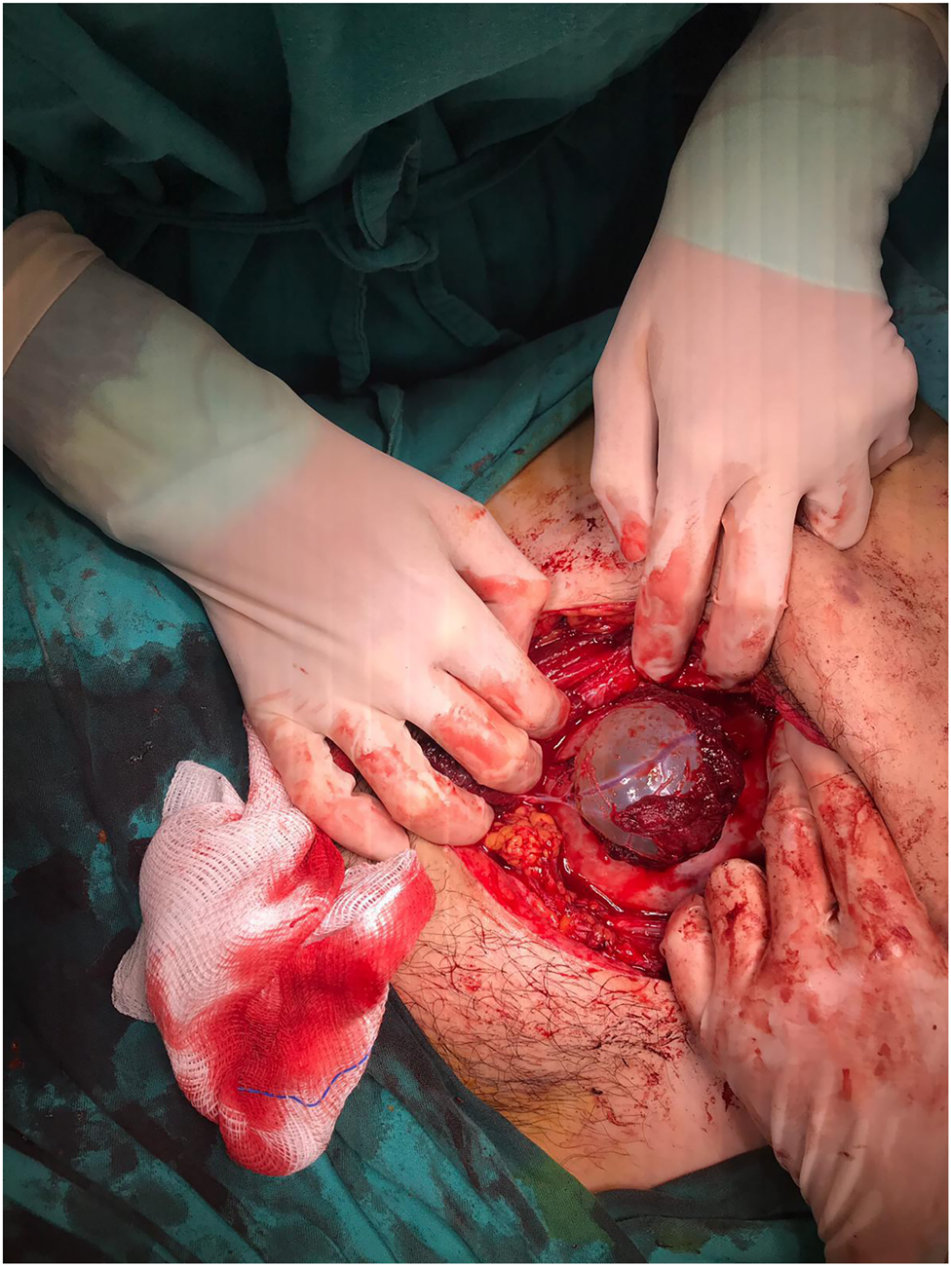
Intraoperative image showing protrusion of amniotic sac through the myometrial defect.

Due to the emergent nature of the condition, the patient and her family were informed that immediate delivery represents the standard management of uterine rupture, and that conservative surgical repair with continuation of pregnancy is a non-standard approach reported only in highly selected cases; following informed consent, diagnostic laparoscopy was performed. During the procedure, widespread coagulum was observed around the uterus. A 4–5 cm full-thickness myometrial defect with an intact amniotic sac was identified. Intraoperatively, the rupture site was found to correspond to the previous uterine scar line. The lower side of the placenta and amniotic sac was protruding from the defect (Figure 3), necessitating conversion to an open surgical approach via a Pfannenstiel incision.

**Figure 4:**
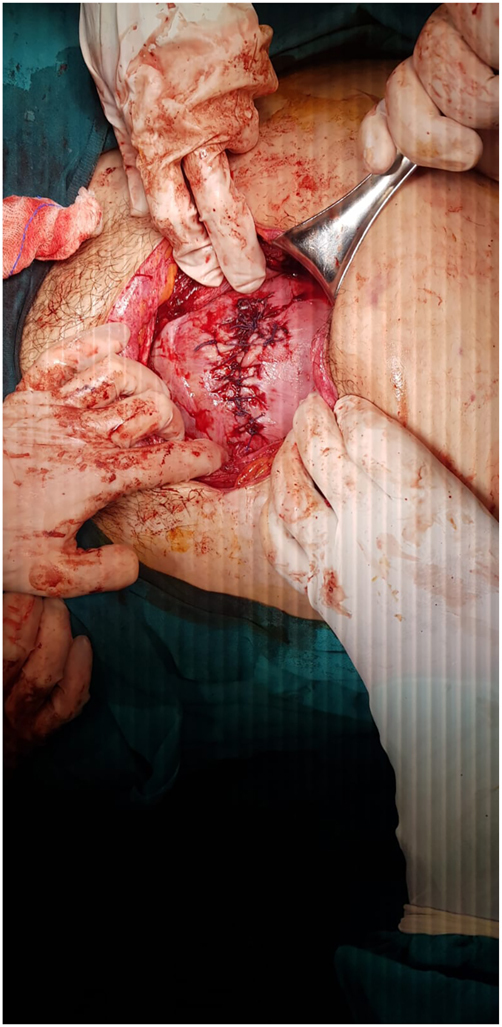
Postoperative image showing the uterine defect after surgical repair.

Upon entering the abdomen, minimal bleeding was observed at the corners of the defect. Maternal vital signs were stable. The fetal heart rate was normal and recorded during the operation. The uterine defect was repaired using 1 Polyglactin 910 (Vicryl) suture (Figure 4). Corner sutures were placed first to control bleeding and maintain anatomical landmarks. Afterwards, the rupture line was closed with simple interrupted stitches, placed from the sides to the middle to reduce tension. Sutures were placed through the full myometrial thickness, while avoiding injury to the amniotic membrane. The second layer was placed using separate imbricating sutures with approximately 1 cm spacing between stitches, and no adjunct hemostatic agents were required.

The intraoperative course was stable, and no blood products were required during the procedure. A postoperative ultrasound confirmed fetal heart rate activity and adequate amniotic fluid.

In the postoperative period, the patient received indomethacin for tocolysis and intravenous cefazolin for 24 h. Given the high risk of preterm delivery in the postoperative period, a full course of antenatal corticosteroids was administered. Due to a drop in hemoglobin levels to 7.7 g/dL following surgery, she received two units of packed erythrocytes and two units of fresh frozen plasma. Postoperatively, the patient was followed with serial ultrasonography to monitor for dehiscence or recurrent rupture. In our institutional practice, a myometrial thickness of ≤0.5 mm is considered suggestive of dehiscence; therefore, follow-up assessments were primarily focused on whether the thickness remained above this critical threshold rather than recording exact numeric values at each examination. Throughout surveillance, the myometrial thickness was consistently evaluated as greater than 0.5 mm. MRI performed 3 weeks post-surgery demonstrated normal uterine wall integrity.

**Figure 5:**
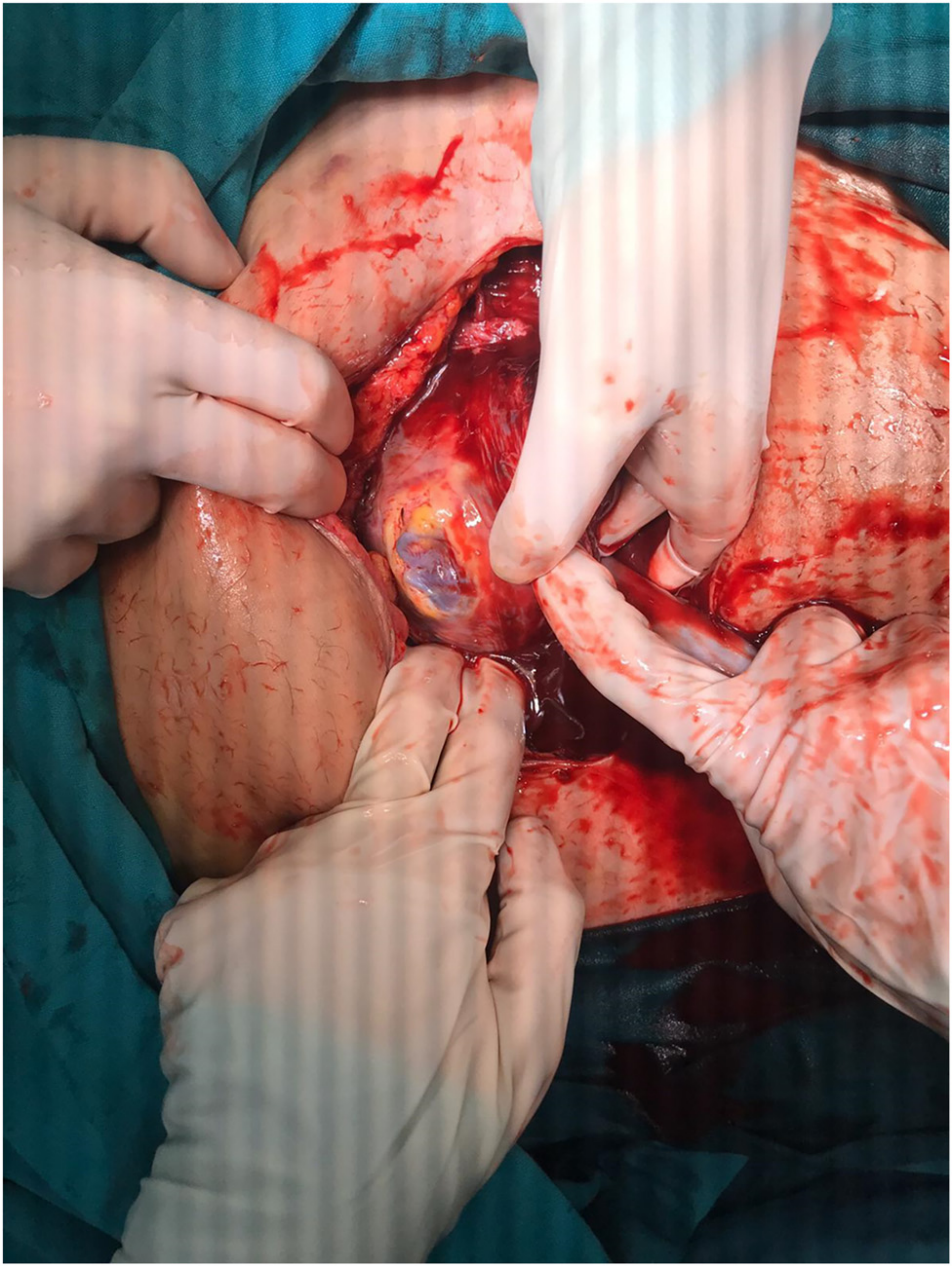
Intraoperative image showing dehiscence occurring at previously repaired rupture area.

**Figure 6:**
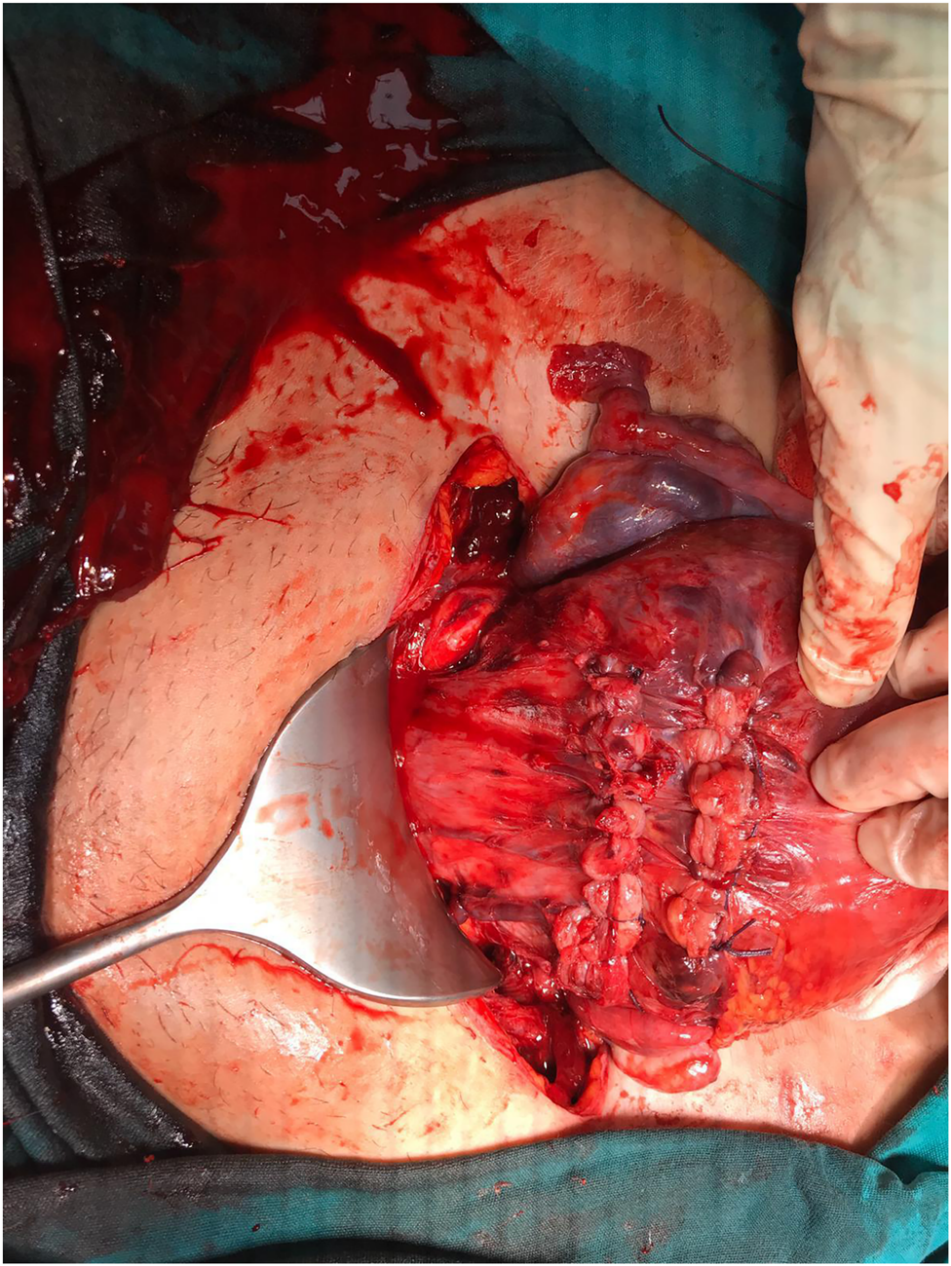
Postoperative image showing repaired cesarean incision and dehiscence area.

Given the risk of recurrent uterine rupture and the non-standard nature of conservative management, the patient was closely monitored as an inpatient until delivery for intensive maternal and fetal surveillance. At 33 weeks and 6 days, she underwent elective cesarean delivery due to contractions and the high risk of rupture. A 3 cm dehiscence was observed at the previously repaired rupture line (Figure 5) during cesarean delivery; this area and the cesarean incision were repaired using 1 Polyglactin 910 (Vicryl) sutures (Figure 6).

Both mother and infant remained stable postpartum, and the mother was discharged on postoperative day 4. The newborn, weighing 2,230 g, initially required NICU monitoring for 3 days due to tachypnea but was later discharged without complications. Long-term follow-up evaluations confirmed that both motor and mental development were consistent with age up to one year post-delivery.

## Discussion

Uterine rupture is defined as disruption of the uterine muscle involving the uterine serosa and is associated with bleeding, fetal compromise, and expulsion of the fetus or placenta into the abdominal cavity. This condition differs from uterine dehiscence, in which the uterine serosa remains intact. Dehiscence is most commonly recognized at cesarean delivery but may also be suspected via ultrasound during pregnancy or even routine gynecologic assessments.

Management of dehiscence presents a significant challenge. While literature has reported successful repair of dehiscence cases [4], the preferred management strategy remains careful monitoring and planned cesarean delivery prior to labor [5]. In our patient, dehiscence was identified at birth; neither fetal nor maternal condition was adversely affected.

Spontaneous uterine rupture, unlike dehiscence, requires urgent intervention. It most commonly occurs during labor in the third trimester. The most significant risk factor is a history of cesarean delivery [6]. Other risk factors include short interpregnancy interval, preterm cesarean, classical incision, placental invasion anomalies, uterine overdistension, and labor induction [7], 8]. Compared with previously reported cases such as those by Hawkins et al. and the review by Sharon et al., our case is notable for rupture at 23 weeks of gestation, successful pregnancy prolongation exceeding 10 weeks, and MRI-supported diagnosis followed by double-layer absorbable repair.

Evidence indicates that early recognition and timely surgical intervention are crucial for reducing morbidity and mortality [9], 10]. Antepartum diagnosis is challenging due to nonspecific signs and symptoms [11], 12]. Sharon et al. (2021) reviewed 18 cases, noting that most presented with severe abdominal pain and altered vitals. We hypothesize that the nonspecific initial symptoms in our case were related to uterine scar tension, with hemoperitoneum developing later.

MRI appears to be a highly effective imaging modality for diagnosing uterine rupture [9], 13]. Historically, management often involved hysterectomy [14], but conservative surgical repair has gained acceptance in selected cases with controlled bleeding and fetal viability [15].

Our case demonstrates that timely surgical repair may allow pregnancy prolongation and improve neonatal outcomes. We used 1 Polyglactin 910 (Vicryl), which maintains 50 % tensile strength for 21 days and is absorbed within 56–70 days – allowing fetal growth toward viability. We preferred absorbable sutures to reduce risks associated with nonabsorbable materials.

Postoperatively, the patient was hospitalized until delivery for close maternal and fetal monitoring. MRI confirmed uterine integrity, and fetal growth was normal. Similar to prior reports [15], individualized care contributed to the successful outcome.

Further research is needed to refine management protocols for spontaneous uterine rupture and improve early diagnosis through imaging and vigilant monitoring. For clinicians, this case highlights that in carefully selected and hemodynamically stable patients, conservative uterine repair with close multidisciplinary surveillance may be considered as an alternative to immediate delivery.
